# Pancreatic cancer with pseudoaneurysm after duckbill‐shaped anti‐reflux metal stent placement: A case report

**DOI:** 10.1002/deo2.203

**Published:** 2022-12-20

**Authors:** Keisuke Kinoshita, Kazuhiro Mizukami, Kensuke Fukuda, Kazuhisa Okamoto, Ryo Ogawa, Tadayoshi Okimoto, Masaaki Kodama, Kazunari Murakami

**Affiliations:** ^1^ Department of Gastroenterology Faculty of Medicine Oita University Oita Japan

**Keywords:** duckbill‐shaped anti‐reflux metal stent, pancreatic cancer, pseudoaneurysm, self‐expandable metal stent, transcatheter arterial embolization

## Abstract

A 74‐year‐old man was diagnosed with unresectable pancreatic cancer with obstructive jaundice. Chemotherapy with gemcitabine and nab‐paclitaxel was initiated after placement of a duckbill‐shaped anti‐reflux metal stent (D‐ARMS). A period of 1 month after D‐ARMS placement, the patient developed hematemesis and entered severe shock following emergency admission for further evaluation.

Contrast‐enhanced computed tomography revealed a pseudoaneurysm in the gastroduodenal artery, coincident with the site of D‐ARMS placement, and bleeding from the same site was diagnosed. Angiography was performed, and the pseudoaneurysm was successfully treated by transcatheter arterial embolization using coils. The patient was subsequently discharged from hospital and experienced no further bleeding until his death due to an aggravation of the pancreatic cancer after 2 months. We report a case of pancreatic cancer with pseudoaneurysm after D‐ARMS placement.

## INTRODUCTION

Endoscopic biliary stenting is the standard therapy for malignant biliary obstruction (MBO). A self‐expandable metal stent (SEMS) is frequently used for treating MBO due to cholangiocarcinoma or pancreatic cancer because of its long patency. However, various adverse events have been reported in association with SEMS, including cholangitis, cholecystitis, pancreatitis, liver abscess, and stent migration. Although the incidence is very rare, a potentially life‐threatening adverse event after SEMS placement is pseudoaneurysm formation. Exact diagnosis is extremely important in such cases. Here, we report a case of pancreatic cancer with pseudoaneurysm after the placement of a duckbill‐shaped anti‐reflux metal stent (D‐ARMS).

## CASE REPORT

**FIGURE 1:**
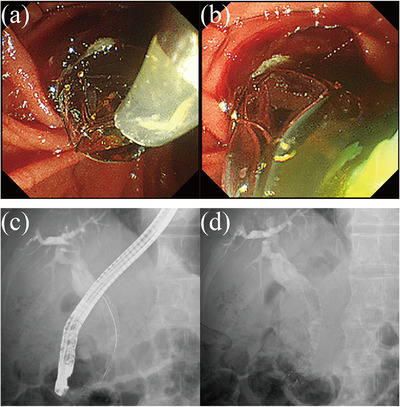
(a) The duckbill‐shaped anti‐reflux metal stent (D‐ARMS) is placed to treat malignant biliary obstruction (MBO) caused by pancreatic cancer. (b) The D‐ARMS is deployed about 1 cm into the duodenal lumen, achieving excellent bile drainage after placement. (c) Endoscopic retrograde cholangiopancreatography findings after D‐ARMS placement. (d) Cholangiography findings after D‐ARMS placement

A 74‐year‐old man with epigastralgia was referred to our hospital after a tumor of pancreatic head was detected on contrast‐enhanced computed tomography (CE‐CT) of the abdomen. Histopathological examination of a specimen from endoscopic ultrasound‐fine needle aspiration of the pancreatic head tumor revealed adenocarcinoma. CE‐CT performed again prior to surgical resection revealed liver metastasis and circumferential invasion of the main trunk of the superior mesenteric artery by pancreatic cancer, which was deemed unresectable. A D‐ARMS was placed to treat MBO due to pancreatic cancer (Figure 1). Chemotherapy with gemcitabine plus nab‐paclitaxel was then initiated.

A period of 1 month after D‐ARMS placement, the patient presented with hematemesis and shock vitals and was admitted to the emergency room. Laboratory data revealed: white blood cell count, 5950/μl; red blood cell count, 206 × 10^4^/μl; hemoglobin, 6.5 g/dl; and platelet count, 33.4 × 10^4^/μl. Serum albumin level was 1.9 g/dl. Blood urea nitrogen level was 24.1 mg/dl. Creatinine level was 1.11 mg/dl. Levels of lactate dehydrogenase, liver enzymes, and amylase were all within normal ranges. The patient was treated with fluid resuscitation and blood transfusion.

**FIGURE 2:**
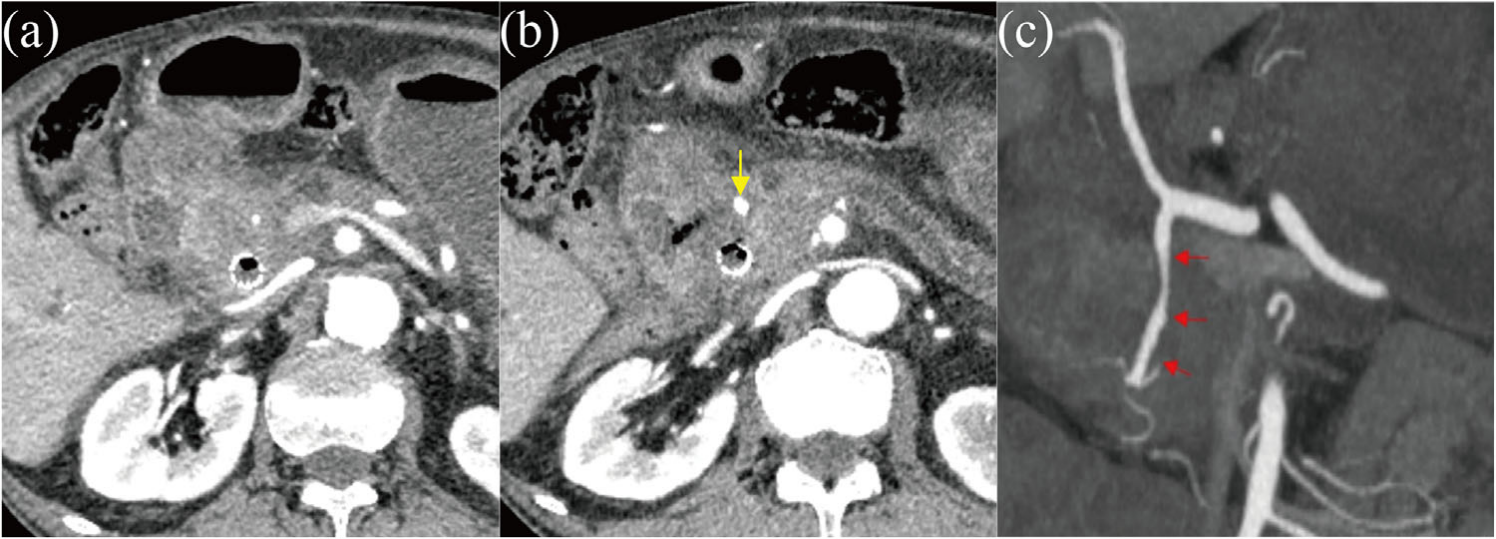
(a) Contrast‐enhanced computed tomography reveals narrowing in the gastroduodenal artery near the duckbill‐shaped anti‐reflux metal stent. (b) Contrast‐enhanced computed tomography reveals dilatation in the peripheral gastroduodenal artery compared to (a), considered to represent a pseudoaneurysm (yellow arrow). (c) Narrowing and irregular dilatation (red arrow) are found in the gastroduodenal artery and determined to represent pseudoaneurysm.

**FIGURE 3:**
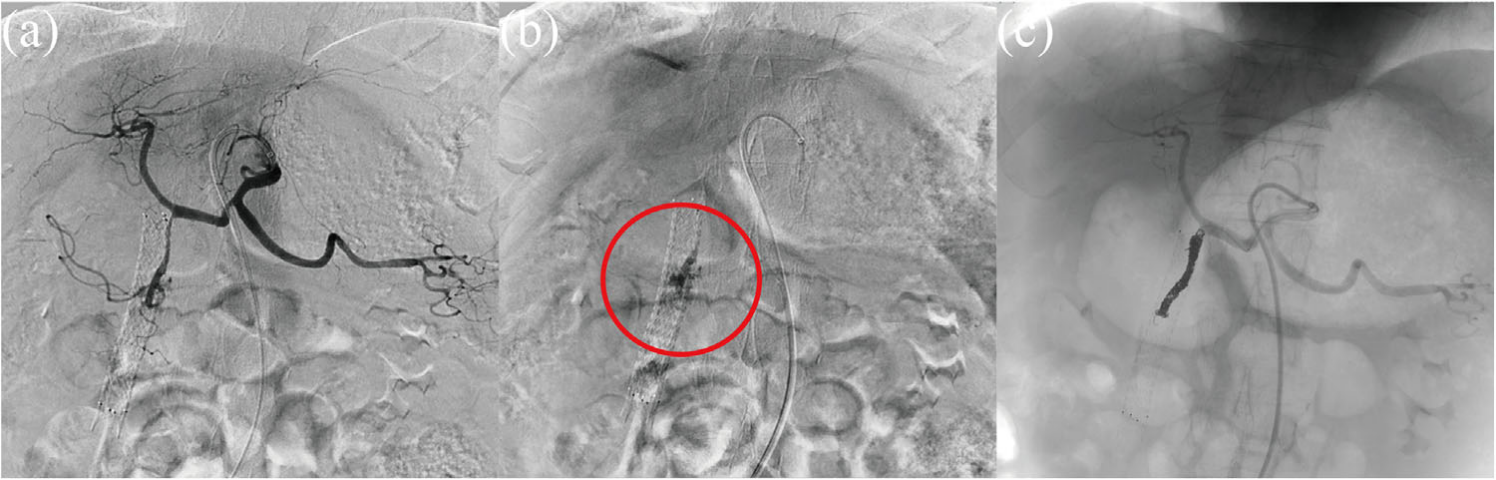
(a) Angiography from the celiac artery reveals narrowing and irregular dilatation in the gastroduodenal artery near the duckbill‐shaped anti‐reflux metal stent. (b) Angiography reveals pseudoaneurysm (red circle) of the gastroduodenal artery. (c) Transcatheter arterial embolization is performed using coils.

CE‐CT revealed a gastroduodenal arterial pseudoaneurysm adjacent to the site of D‐ARMS placement, which had likely ruptured and caused the hematemesis (Figure 2). Emergency angiography was performed. Angiography from the celiac artery revealed narrowing and irregular dilatation from the gastroduodenal artery to the anterior superior pancreaticoduodenal artery, which appeared to be the pseudoaneurysm seen on CE‐CT, so transcatheter arterial embolization (TAE) with coils was performed (Figure 3). After TAE, the patient's vitals stabilized. He was subsequently discharged from our hospital after 2 weeks without experiencing any further bleeding. The patient continued chemotherapy with gemcitabine plus nab‐paclitaxel but died 2 months later due to an aggravation of pancreatic cancer.

## DISCUSSION

SEMS for MBO offers longer patency than plastic stents and has recently been reported as useful for preventing stent occlusion in patients undergoing neoadjuvant chemotherapy (NAC) for pancreatic cancer a relatively long time, 2–3 months before surgery.^1^ Nevertheless, cases of recurrent biliary obstruction (RBO) due to sludge formation or food impaction have been reported after SEMS placement. Recently, the efficacy and safety of biliary drainage using D‐ARMS for MBO has been reported.^2^, ^3^, ^4^ Hinokuchi *et al.* retrospectively compared the incidence of RBO after stent placement between D‐ARMS and conventional covered‐SEMS (CSEMS) in patients undergoing NAC for pancreatic cancer and reported the efficacy and safety of D‐ARMS.^4^ Although our patient had unresectable pancreatic cancer, no RBO was encountered after D‐ARMS placement and biliary drainage during the procedure was excellent. However, no cases of pancreatic cancer with pseudoaneurysm after D‐ARMS placement have previously been described.

Complications associated with SEMS placement have been reported to include cholangitis, cholecystitis, pancreatitis, liver abscess, and stent migration. Pseudoaneurysm formation is a very rare complication of SEMS placement, but the potentially life‐threatening nature of this event makes accurate diagnosis and treatment extremely important. CE‐CT followed by angiography is effective for the diagnosis of pseudoaneurysm. Correct diagnosis of pseudoaneurysm from endoscopy, on the other hand, may prove difficult. In our case, diagnosis of the pseudoaneurysm by endoscopy was difficult. The first‐line treatment for pseudoaneurysm would common to try endoscopic hemostasis, such as medial CSEMS placement, and if hemostasis cannot be achieved, the next strategy of hemostasis by angiography may be considered. Widely used in splenic and hepatic pseudoaneurysms among abdominal pseudoaneurysms, TAE can be performed following angiography to avert rupture and subsequent fatal bleeding. Itoi *et al.* recently reported the efficacy of CSEMS placement as a treatment for biliary hemorrhage.^5^ In our case, because the patient had already undergone D‐ARMS placement and had unresectable pancreatic cancer, we were able to stop the bleeding by prioritizing a less‐invasive endovascular treatment instead of further CSEMS placement or emergency surgery.

The timing of pseudoaneurysm formation has been reported to vary from 5 days to within 1 year after SEMS placement.^6^, ^7^, ^8^ All except one patient among the described cases had received chemotherapy or radiotherapy. Our patient had also received chemotherapy with gemcitabine plus nab‐paclitaxel, which may have weakened the bile duct wall. Pseudoaneurysms frequently formed in the right hepatic artery near the upper end of the SEMS due to its anatomical location at the intersection of the common bile duct (CBD) and the right hepatic artery. In our case, the tip of the D‐ARMS was placed about 1 cm into the duodenal lumen, and the upper end of the D‐ARMS was not placed at the intersection of the CBD and the right hepatic artery. Rather, the pseudoaneurysm formed in the gastroduodenal artery near the site of D‐ARMS placement. The locations of pseudoaneurysm formation of the other five cases were different from the right hepatic artery near the SEMS edge to the gastroduodenal artery in the middle of the SEMS, respectively (Table 1). This anatomical difference may reflect different mechanisms responsible for causing pseudoaneurysms.

**TABLE 1:** Patient demographics, clinical manifestation, type of stent, treatment, and prognosis

Authors, (ref)	Age (years/sex)	Symptoms	Antineoplastic therapy	Time to bleeding (days)	Type of stent	Length of stent (mm)	Diameter of stent (mm)	Bleeding focus	Treatment	Prognosis
Hyun *et al.* ^6^	61/M	Melena	Chemotherapy	152	N/A	50–100	10	GDA	TAE	No rebleeding
	51/M	Melena	Chemotherapy	76	N/A	50–100	10	GDA	TAE	No rebleeding
Nezu *et al.* ^7^	72/M	Hematemesis and melena	CRT	5	Covered Wallstent	60	10	RHA	None	Died
	82/F	Hematemesis	None	20	Uncovered Wallflex	60	10	PSPDA	TAE	No rebleeding
Fujimori *et al.* ^8^	65/F	Hemobilia	Chemotherapy	14	Covered Hanarostent	80	10	GDA	Covered SEMS replacement and TAE	No rebleeding
Our case	74/M	Hematemesis	Chemotherapy	28	D‐ARMS	80	10	GDA	TAE	No rebleeding

Abbreviations: CRT, chemoradiation therapy; D‐ARMS, duckbill‐shaped anti‐reflux metal stent; F, female; GDA, gastroduodenal artery; M, male; N/A, not available; PSPDA, posterior superior pancreaticoduodenal artery; RHA, right hepatic artery; SEMS, self‐expandable metallic stent; TAE, transarterial embolization.

The mechanism of pseudoaneurysm formation following SEMS placement is thought to involve tumor progression and invasion of the artery, inflammation and weakening of the artery and bile duct walls due to cholangitis or chemoradiotherapy, with physical pressure from the radial force of the SEMS or metal wires at the SEMS tip causing necrosis in the adjacent artery, leading to perforation and bleeding into the bile duct with weak resistance. The diameter of the CBD may be also one of the important factors causing pseudoaneurysms. In our case, the diameter of CBD had 8 mm, but a 10‐mm‐diameter D‐ARMS was used. This discrepancy between the diameter of the CBD and the SEMS may be also thought to be one of the factors causing pseudoaneurysm. The most popular SEMSs have 8‐ or 10‐mm‐diameter and can easily be deployed in a dilated CBD. Although, either an 8‐ or 10‐mm‐diameter SEMS may be relatively large for the obstructed CBD which has a lumen collapsed like a pinhole and surrounded by tumor when the SEMS is deployed in its original diameter.^6^ Only six cases of pancreatic cancer with pseudoaneurysm after SEMS placement have been reported, including our case, and only our case used a laser‐cut‐type D‐ARMS.^6^, ^7^, ^8^ In addition, a 10‐mm‐diameter SEMS was used in all of these cases, including our case (Table 1). This suggests that the use of SEMS with a large diameter of 10 mm and strong radial force may be a factor in the formation of pseudoaneurysms, along with characteristic complications, such as cholecystitis and pancreatitis. Based on the above, the bile duct wall and blood vessels may have become fragile due to physical pressure from the radial force of the D‐ARMS on the adjacent artery, the discrepancy between the diameter of the CBD and the SEMS, the history of multiple placements of bile duct stents, long‐term use of immunosuppressive drugs, and invasion by the pancreatic head cancer, contributing to the formation of the pseudoaneurysm in our case. In the event of similar cases in the future, we will pay attention to each endoscopic manipulation during stent exchanges and other procedures.

In this report, we have described a case of pancreatic cancer with pseudoaneurysm after D‐ARMS placement for MBO, in which hemostasis was successfully achieved with TAE to save the patient's life. Although early diagnosis of pseudoaneurysm can be difficult, pseudoaneurysm formation needs to be kept in mind as a complication after SEMS placement in future treatment and diagnosis in the biliopancreatic region.

## CONFLICT OF INTERESTS

None.

## ETHICS STATEMENT

All procedures were performed in accordance with the ethical standards laid down in the 1964 Declaration of Helsinki and its later amendments.

## References

[1] Kubota K , Sato T , Watanabe S *et al.* Covered self‐expandable metal stent deployment promises safe neoadjuvant chemoradiation therapy in patients with borderline resectable pancreatic head cancer. Dig Endosc 2014; 26: 77–86. 23551230 10.1111/den.12049

[2] Kin T , Ishii K , Okabe Y , Itoi T , Katanuma A . Feasibility of biliary stenting to distal malignant biliary obstruction using a novel designed metal stent with duckbill‐shaped anti‐reflux valve. Dig Endosc 2021; 33: 648–55. 32875614 10.1111/den.13827

[3] Yamada Y , Sasaki T , Takeda T *et al.* A novel laser‐cut fully covered metal stent with anti‐reflux valve in patients with malignant distal biliary obstruction refractory to conventional covered metal stent. J Hepatobiliary Pancreat Sci 2021; 28: 563–71. 33835728 10.1002/jhbp.966

[4] Hinokuchi M , Hashimoto S , Kojima I *et al.* Efficacy and safety of a novel anti‐reflux metal stent during neoadjuvant chemotherapy for pancreatic cancer: A prospective multicenter exploratory study. J Hepatobiliary Pancreat Sci Published online: 15 Sep 2022; DOI: 10.1002/jhbp.1239 36106919

[5] Itoi T , Yasuda I , Doi S , Mukai T , Kurihara T , Sofuni A . Endoscopic hemostasis using covered metallic stent placement for uncontrolled post‐endoscopic sphincterotomy bleeding. Endoscopy 2011; 43: 369–72. 21360425 10.1055/s-0030-1256126

[6] Hyun D , Park KB , Hwang JC , Shin BS . Delayed, life‐threatening hemorrhage after self‐expandable metallic biliary stent placement: Clinical manifestations and endovascular treatment. Acta Radiol 2013; 54: 939–43. 23761546 10.1177/0284185113485501

[7] Nezu Y , Nakaji S , Fujii H , Ishii E , Hirata N . Pseudoaneurysm caused by a self‐expandable metal stent: A report of three cases. Endoscopy 2014; 46: 248–51. 24573734 10.1055/s-0033-1359178

[8] Fujimori N , Matsumoto K , Murakami M , Suehiro Y , Oono T . Endoscopic tamponade using a fully covered self‐expandable metallic stent for massive biliary bleeding from a pseudoaneurysm rupture during metallic stent removal. VideoGIE 2021; 6: 24–6. 33490750 10.1016/j.vgie.2020.08.009 PMC7804991

